# Key circular RNAs identified in male osteoporosis patients by whole transcriptome sequencing

**DOI:** 10.7717/peerj.11420

**Published:** 2021-05-26

**Authors:** Haijin Zhang, Xue Song, Zongyan Teng, Sujun Cheng, Weigang Yu, Xiaoyi Yao, Zhiqiang Song, Yina Zhang

**Affiliations:** 1 Department of Geriatrics, The Second Affiliated Hospital of Harbin Medical University, Harbin, China; 2 Harbin North people’s Hospital, Harbin, China

**Keywords:** Whole transcriptome sequencing, Male osteoporosis, Non-coding RNAs, circRNAs

## Abstract

**Background:**

Osteoporosis (OP) is a systemic disease with bone loss and microstructural deterioration. Numerous noncoding RNAs (ncRNAs) have been proved to participate in various diseases, especially circular RNAs (circRNAs). However, the expression profile and mechanisms underlying circRNAs in male osteoporosis have not yet been explored.

**Methods:**

The whole transcriptome expression profile and differences in mRNAs, circRNAs, and microRNAs (miRNAs) were investigated in peripheral blood samples of patients with osteoporosis and healthy controls consisting of males ≥ 60-years-old.

**Results:**

A total of 398 circRNAs, 51 miRNAs, and 642 mRNAs were significantly and differentially expressed in osteoporosis compared to healthy controls. Gene ontology (GO) and Kyoto Encyclopedia of Genes and Genomes (KEGG) enrichment analysis showed that the host genes of significantly differentially expressed circRNAs were mainly enriched in the regulation of cell cycle process: biological process (BP), organelle part cellular components (CC), protein binding molecular function (MF), Toll-like receptor signaling pathway, tumor necrosis factor (TNF) signaling pathway, and thyroid hormone signaling pathway. circRNA-miRNA-mRNA regulatory network was constructed using the differentially expressed RNAs. Moreover, key circRNAs (hsa_circ_0042409) in osteoporosis were discovered and validated by qPCR.

**Conclusions:**

The key cicrRNAs plays a major role in the pathogenesis of osteoporosis and could be used as potential biomarkers or targets in the diagnosis and treatment of osteoporosis.

## Introduction

Osteoporosis (OP) is a systemic disease with osteopenia and microstructural deterioration that increases the risk of fracture susceptibility, especially in the spine, buttocks, and wrists (Kuo & Chen, 2017). Male osteoporosis is a common age-related degenerative disease, characterized by impaired bone formation and low bone turnover. Postmenopausal osteoporosis is related to excessive bone resorption caused by estrogen deficiency. According to the report of the World Health Organization (WHO), the number of OP patients is increasing rapidly (Cabral et al., 2016), and accounts for about 6.6% of the total population in China (Zhang et al., 2018a). Men have a higher mortality rate after fracture than women (Bliuc & Center, 2016). Previous studies have identified some biochemical indexes of OP, which can be used for the diagnosis and monitoring of patients with OP (Wang et al., 2017), such as serum collagen type I N-terminal pre-peptide (PINP) and cross-linked C-terminal peptide (CTX) (Yang et al., 2019). However, conventional biochemical markers are not effective in determining the possible secondary causes of osteoporosis in men (Fink et al., 2016), while currently available biochemical markers cannot detect all risk factors for fractures (Cheng et al., 2017). Therefore, it is identifying new biomarkers to improve the diagnosis and treatment of osteoporosis in men is imperative.

Accumulating evidence shows that ncRNAs are associated with various diseases through indirect or direct regulation of the corresponding gene expression (Yang et al., 2018). ncRNAs, including miRNA and circRNA, play a crucial role in the occurrence, development, and progression of cancer (Dou et al., 2016). Importantly, previous studies have suggested that circulating miRNAs may be used as a critical biomarker for osteoporosis (Fu et al., 2018; Materozzi et al., 2018).

circRNAs are a class of endogenous, abundant, non-polyadenylated RNAs with a covalently closed, continuous loop structure (Zhang, Yang & Xiao, 2018b). These RNAs are associated with various biological processes, and their dysregulated expression are implicated in human diseases, including diabetes, Alzheimer’s disease, tumors, and cardiovascular disease because of their high stability and prevention from RNA exonuclease degradation (Qiao et al., 2018; Zhao et al., 2017). Interestingly, the complex regulatory interactions between different types of ncRNAs have fundamental roles in the development of multiple diseases (Peng et al., 2018; Zhong et al., 2018). circRNAs are well-known as miRNA sponge in inhibiting the function of miRNA via competing endogenous RNA (ceRNA) network (Zhong et al., 2018). For instance, circRNA-ZNF609, containing multiple binding sites for miR-150-5p, regulates ATK3 expression in Hirschsprung’s disease through ceRNA network (Fu et al., 2018). It has also been proved that circRNA MYLK binds competitively to miRNA29a-3p, thereby increasing the expression of the target genes *VEGFA*, *DNMT3B*, and *ITGB1*, involved in the progression of bladder cancer (Fu et al., 2017). Together, these findings suggested that mRNAs, miRNAs, and circRNAs play a major role in various human diseases, such as osteoporosis (Mandourah et al., 2018).

In the present study, whole transcriptome sequencing was carried out on monocytes from male healthy controls and osteoporosis patients. Key circRNAs involved in the pathogenesis of osteoporosis were identified by bioinformatics analysis. Thus, our findings provide a basis for further in-depth study of pathogenic genes and the rapid, simple diagnosis, and treatment of osteoporosis in men.

## Material and Methods

### Patients and samples

Study participants were collected from October 2016 to November 2017, and bone mineral density (BMD) was examined in the Second Affiliated Hospital of Harbin Medical University. All peripheral blood samples, including healthy controls and patients with osteoporosis, were collected. Healthy controls were defined by spine BMD T-score ≥−1.0 SD, while osteoporosis was defined by spine BMD T-score ≤−2.5 SD. All participants were males, aged ≥ 60-years-old. The detailed characteristics of the study samples are shown in Table 1. This study was approved by the Ethics Committee of The Second Affiliated Hospital of Harbin Medical University (#KY2016-198). All patient samples were obtained at the time of diagnosis, and informed consent was signed at the The Second Affiliated Hospital of Harbin Medical University.

### RNA isolation and RNA sequencing

**Figure 1:**
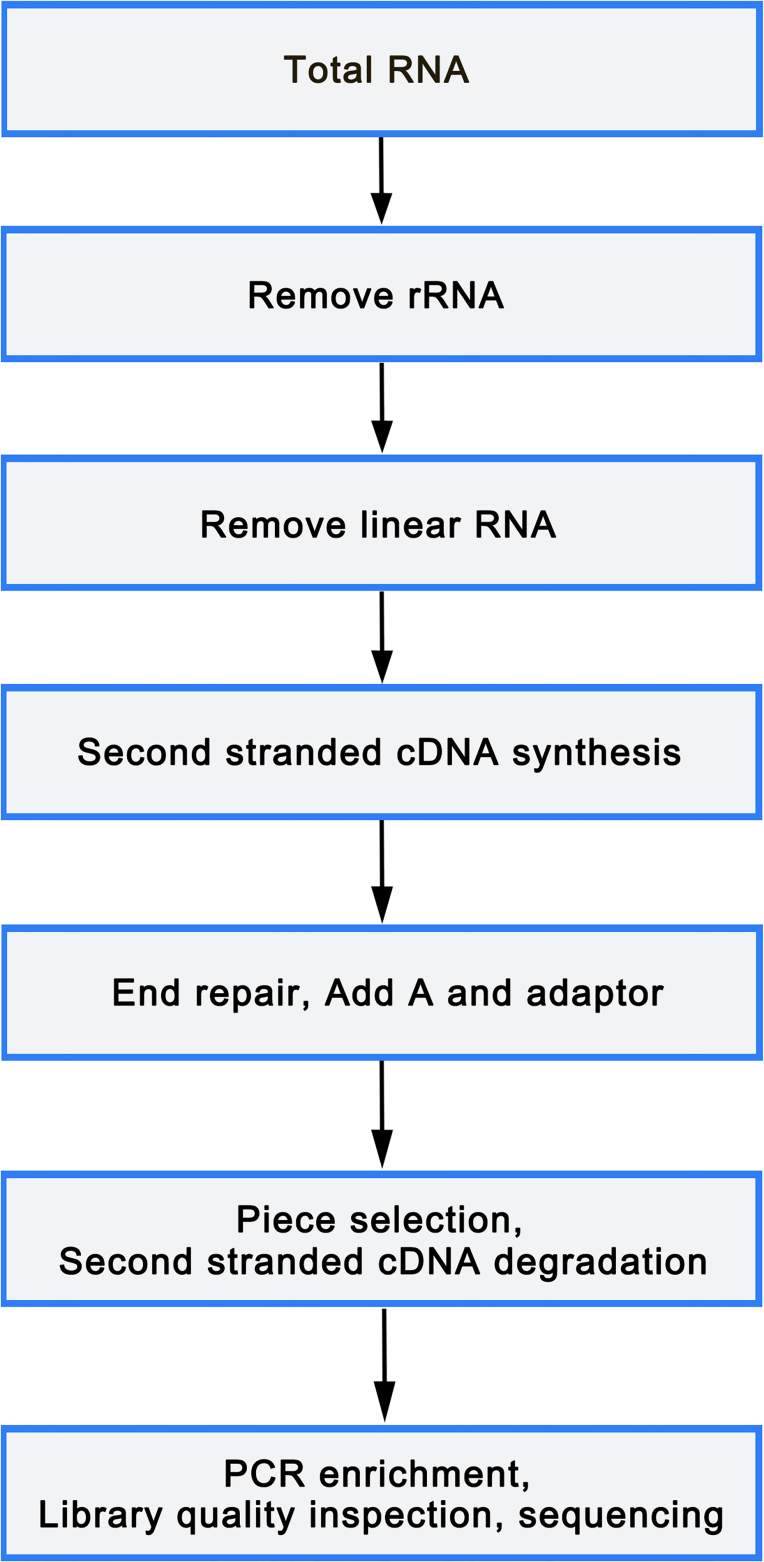
Library sequencing process.

Total RNA was isolated from mononuclear cells of 6 peripheral blood samples (3 OP and 3 healthy controls) using TRIzol reagent (Sigma, St. Louis, USA), following manufacture’s protocol. An equivalent of 5 µg RNA was utilized as input material for the RNA sample preparations. Libraries were constructed utilizing rRNA depleted and RNase R digested RNAs or NEBNext Multiplex Small RNA Library Prep Set for Illumina (NEB, USA), according to manufacturer’s instructions. After cluster generation on a cBot Cluster Generation System using TruSeq PE Cluster Kit v3-cBot or TruSeq SR Cluster Kit v3-cBot-HS (Illumina), the library preparations were sequenced on an Illumina HiSeq 2500/4000 platform. The flowchart was as follows(Fig. 1).

### Bioinformatics analysis

**Figure 2:**
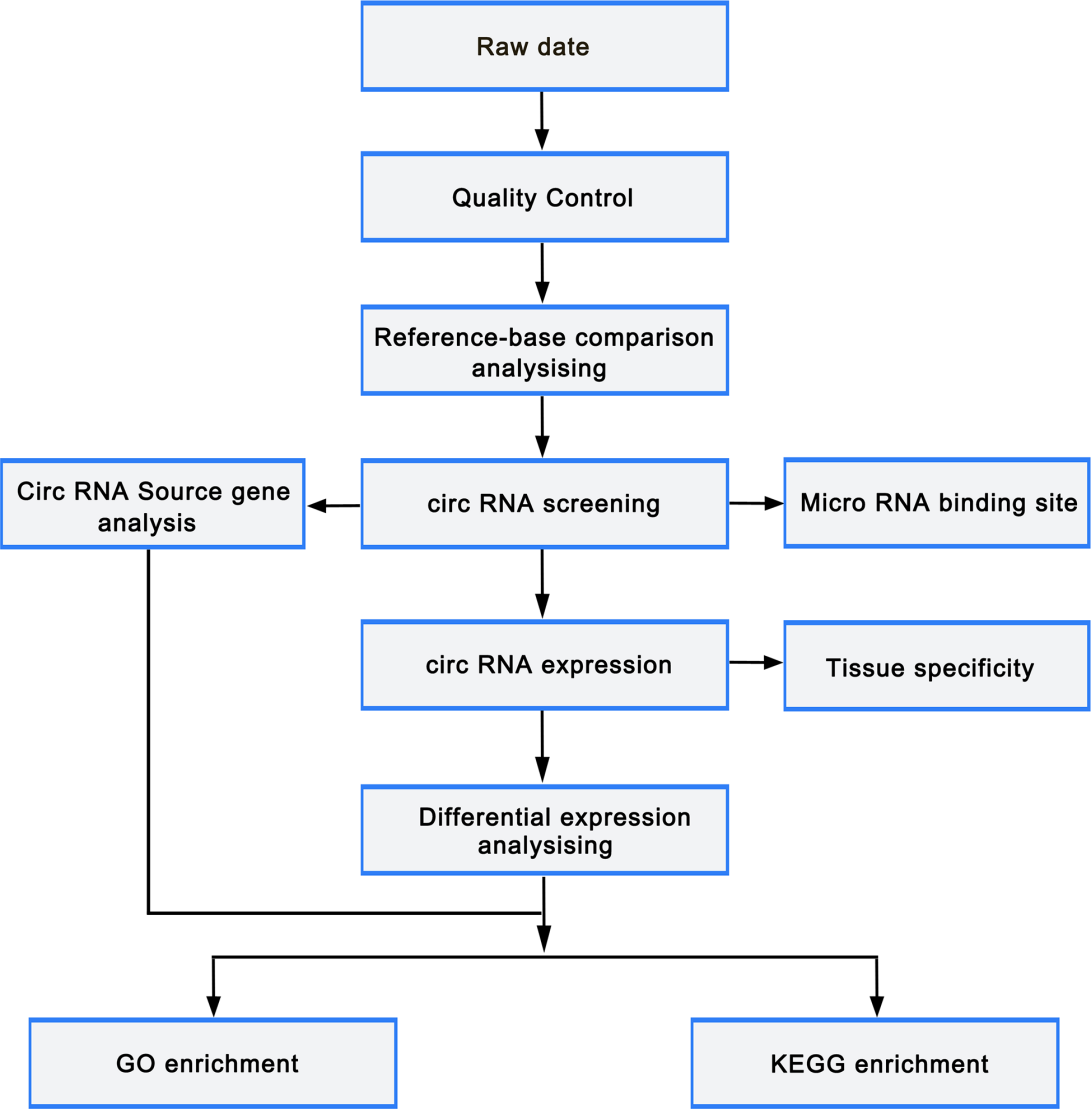
Bioinformatic analysis process.

circRNAs were predicted using find_circ (Memczak et al., 2013) and CIRI2 (Zeng et al., 2017) to reduce false positives. The predicted circRNA results of the two software were intersected based on the position of circRNAs on chromosome. Stringent filter criteria were applied to select candidate circRNAs as follows: at least junction reads ≥5 in one samples or junction reads ≥2 in all samples of one group. The gene expression level was quantified using TPM (readCount ×1,000,000)/libsize. Deseq2 (Love, Huber & Anders, 2014) was employed to perform differentially expressed gene analysis with the cutoff fold-change > 1 and adjust *p*-value < 0.05. GOseq and KOBAS (Xie et al., 2011) were used to carry out Gene ontology (GO) and Kyoto Encyclopedia of Genes and Genomes (KEGG) pathway enrichment analysis, respectively. miRTarBase was used to predict the miRNAs that target the differentially expressed mRNAs, while miRanda was utilized to predict the binding sites of miRNA and circRNA. Cytoscape was employed to construct the miRNA-circRNA-mRNA regulatory network. The flowchart was as follows (Fig. 2).

**Table 1:** Characteristics of the study participants.

Characteristics	OP (*n* = 12)	Control (*n* = 12)	*P* value
Age (year)	62.67 ± 1.61^b^	62.42 ± 1.31	0.68
Height (cm)	173.25 ± 2.86^b^	172.50 ± 2.47	0.50
Weight (kg)	70.33 ± 2.19^b^	71.00 ± 2.09	0.45
BMI (kg/m^2^)	23.43 ± 0.42^b^	23.86 ± 0.63	0.60
Waist (cm)	81.58 ± 2.61^b^	80.08 ± 3.26	0.23
L1-4 BMD (g/cm^2^)	0.76 ± 0.05^a^	1.06 ± 0.03	0.00
C reaction protein (mg/L)	7.76 ± 1.79^b^	6.29 ± 1.89	0.64
Alkaline phosphatase (U/L)	84.58 ± 10.72^a^	68.17 ± 10.25	0.00
25 hydroxyvitamin D (ng/ml)	73.03 ± 18.82^a^	118.94 ± 27.22	0.00
P1NP (ng/ml)	38.03 ± 9.52^a^	59.65 ± 10.28	0.00
CTX (ng/ml)	7.30 ± 1.17^a^	5.05 ± 1.36	0.00

**Notes.**

a *P* < 0.05.

b *P* > 0.05.

### Quantitative real-time PCR validation

Quantitative real-time PCR (qRT-PCR) evaluated the gene expression in new twelve pairs of samples. The relative expression of mRNA or circRNA was determined by normalization against that of glyceraldehyde 3-phosphate dehydrogenase (*GAPDH*). U6 was employed as an internal control of miRNAs. The primer sequences are as follows: circ_0042409, forward: 5′-CGAGAATCTGAGCCTGAACC-3′, reverse: 5′-GTGGCTGTCCTGCTACTTGA-3′; hsa-miR-195-5p, forward: 5′-TAGCAGCACAGAAATATTGGC-3′, reverse: 5′-GCAGGGTCCGAGGTATTC-3′; *KLC1*, forward: 5′-TCAATGACCCTGAGAACA-3′, reverse: 5′-CTCATACTCACTTCCTCCC-3′.

### Statistical analysis

qRT-PCR experiment was repeated three times. SPSS was utilized for statistical analysis with independent *t*-test. *P* < 0.05 was considered as statistically significant.

## Results

### Differentially expressed circRNAs, miRNAs and mRNAs

Raw data(raw reads) of fastq format were processed using in-house Perlscripts. Clean data (clean reads) were obtained by removing the reads containing adapter, reads containing ploy-N, and low quality reads from raw data. Differential expression analysis of the two groups was performed using the DESeq R package (1.10.1), which further determined the differential expression in digital gene expression data using a model based on the negative binomial distribution. The resulting *P*-values were adjusted using the Benjamini and Hochberg’s approach for controlling the false discovery rate. Genes with an adjusted *P*-value identified by DESeq were termed as differentially expressed.

**Figure 3:**
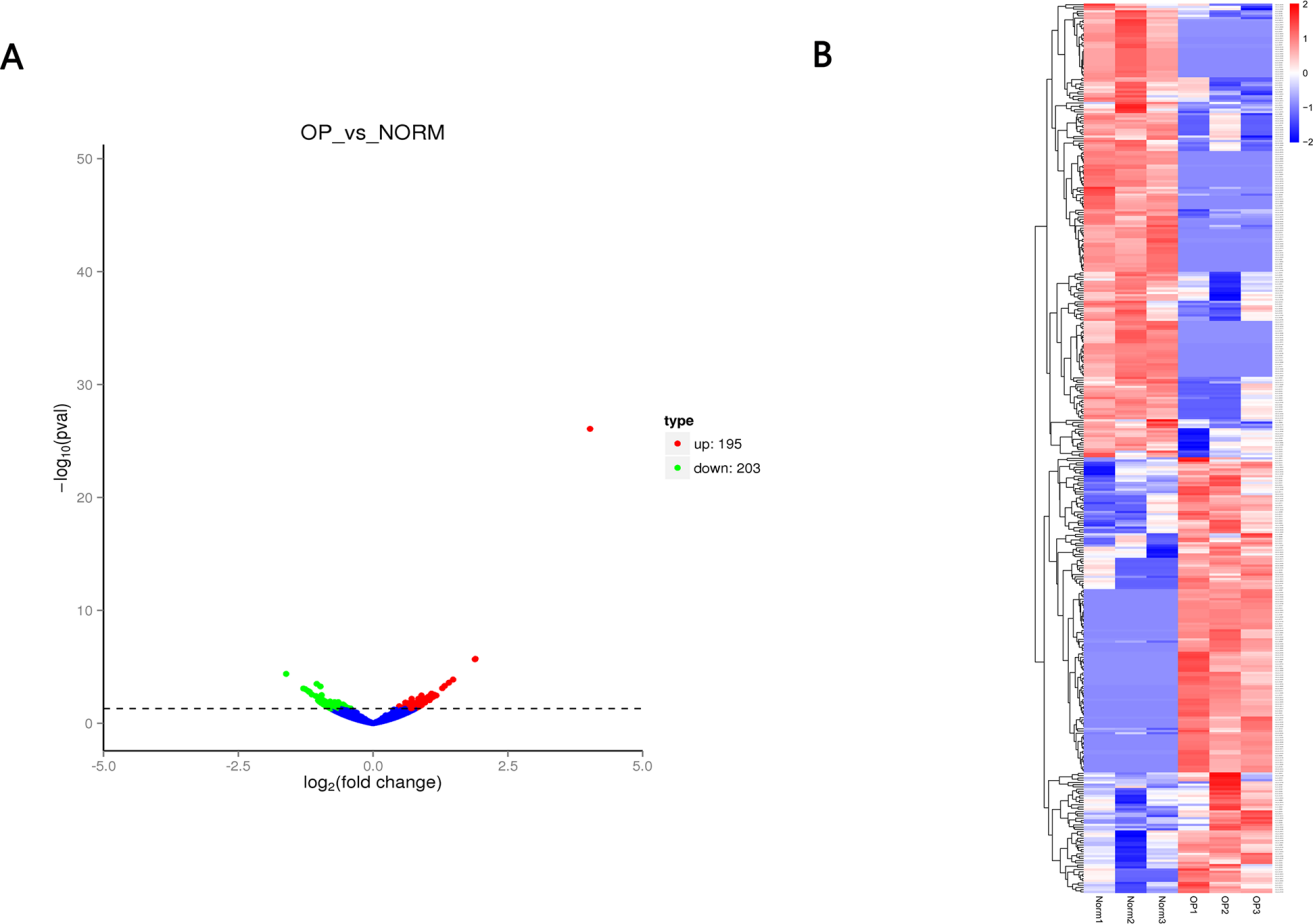
(A) Volcano plot (A) of differentially expressed circRNAs between OP and healthy control. Red indicates upregulated while green represents downregulated. (B) Heatmap of differentially expressed circRNAs between OP and healthy control with red denoting hig expression and blue signifying low expression.

A total of 12,839 circRNAs, including 5,682 circRNAs, were novel. 398 circRNAs were differentially expressed between OP and healthy control. Of those, 195 circRNAs were upregulated while 203 circRNAs were downregulated (Fig. 3 and Table 2) with the cutoff fold-change > 1 and adjusted *P*-value (padj) < 0.05. We also identified 642 differentially expressed mRNAs (305 upregulated and 337 downregulated) and 51 miRNAs (28 upregulated and 23 downregulated), respectively.

**Table 2:** Differentially expressed circRNAs.

ID	log2FC	Pval	Regulation
hsa_circ_0004276	5.1747	1.55E−49	up
hsa_circ_0003060	4.0244	8.44E−27	up
hsa_circ_0005657	1.8979	1.95E−06	up
novel_circ_0020485	1.8896	2.15E−06	up
hsa_circ_0017615	1.4847	0.000134	up
hsa_circ_0004846	1.4061	0.000253	up
novel_circ_0000968	1.3275	0.000491	up
novel_circ_0003426	1.2845	0.000787	up
hsa_circ_0006132	1.1281	0.002859	up
hsa_circ_0042409	1.1104	0.004767	up
novel_circ_0035291	−1.6125	4.20E−05	down
novel_circ_0048949	−1.0447	0.000322	down
novel_circ_0015289	−1.2924	0.000825	down
novel_circ_0006342	−1.2574	0.000944	down
hsa_circ_0000378	−1.1359	0.002834	down
novel_circ_0038918	−1.0059	0.011125	down
novel_circ_0039344	−1.1813	0.001678	down
hsa_circ_0046964	−1.0626	0.003848	down
hsa_circ_0007976	−1.204	0.001402	down
hsa_circ_0003990	−1.0062	0.008878	down

### Functional enrichment analysis of differentially expressed circRNAs

**Figure 4:**
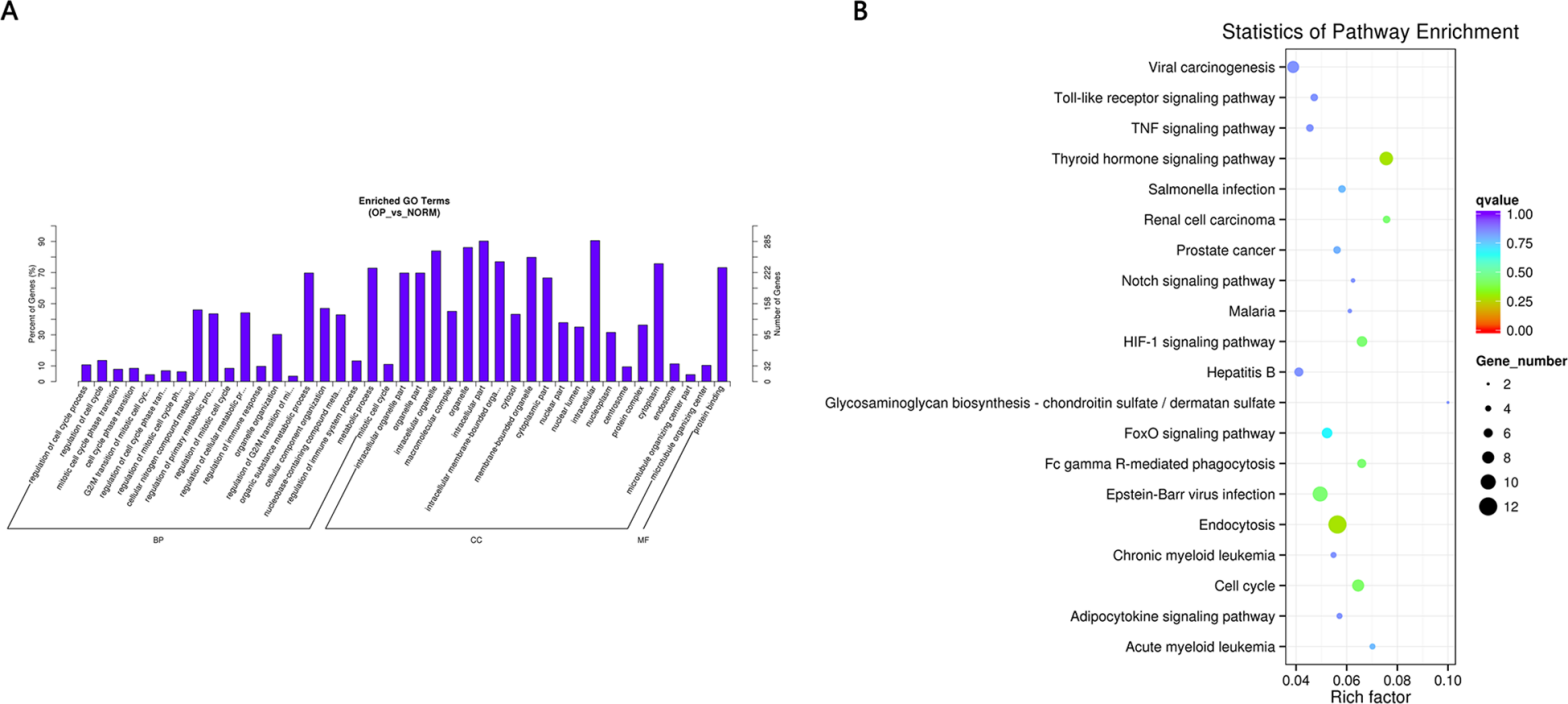
(A) GO enrichment (BP, CC, MF) of host genes of significantly differentially expressed circRNAs. *X*-axis represents the enriched GO term ordered by BP, CC, MF. *Y*-axis indicates the percent (Left) and number (Right) of the genes in the corresponding terms. (B) KEGG enrichment of host genes of significantly differentially expressed circRNAs with color reflecting q-value and node size denoting the number of gene in each pathway.

GO and KEGG pathway enrichment analysis were carried out using the host genes of significantly differentially expressed circRNAs. These circRNAs are mainly enriched in biological processes (BP), including metabolic process (GO:0008152), cellular metabolic process (GO:0044237), biological regulation (GO:0065007) and regulation of cellular process (GO:0050794); cellular component (CC) like intracellular organelle (GO:0043229) macromolecular complex (GO:0032991), organelle (GO:0043226) and membrane-bounded organelle (GO:0043227); and molecular function (MF) including binding (GO0005488) and protein binding (GO:0005515) (Fig. 4A and Table 3). The differentially expressed circRNAs were also enriched in viral carcinogenesis, Toll-like receptor signaling pathway, tumor necrosis factor (TNF) signaling pathway, and thyroid hormone signaling pathway (Fig. 4B and Table 4).

### circRNA-miRNA-mRNA regulatory network construction

**Figure 5:**
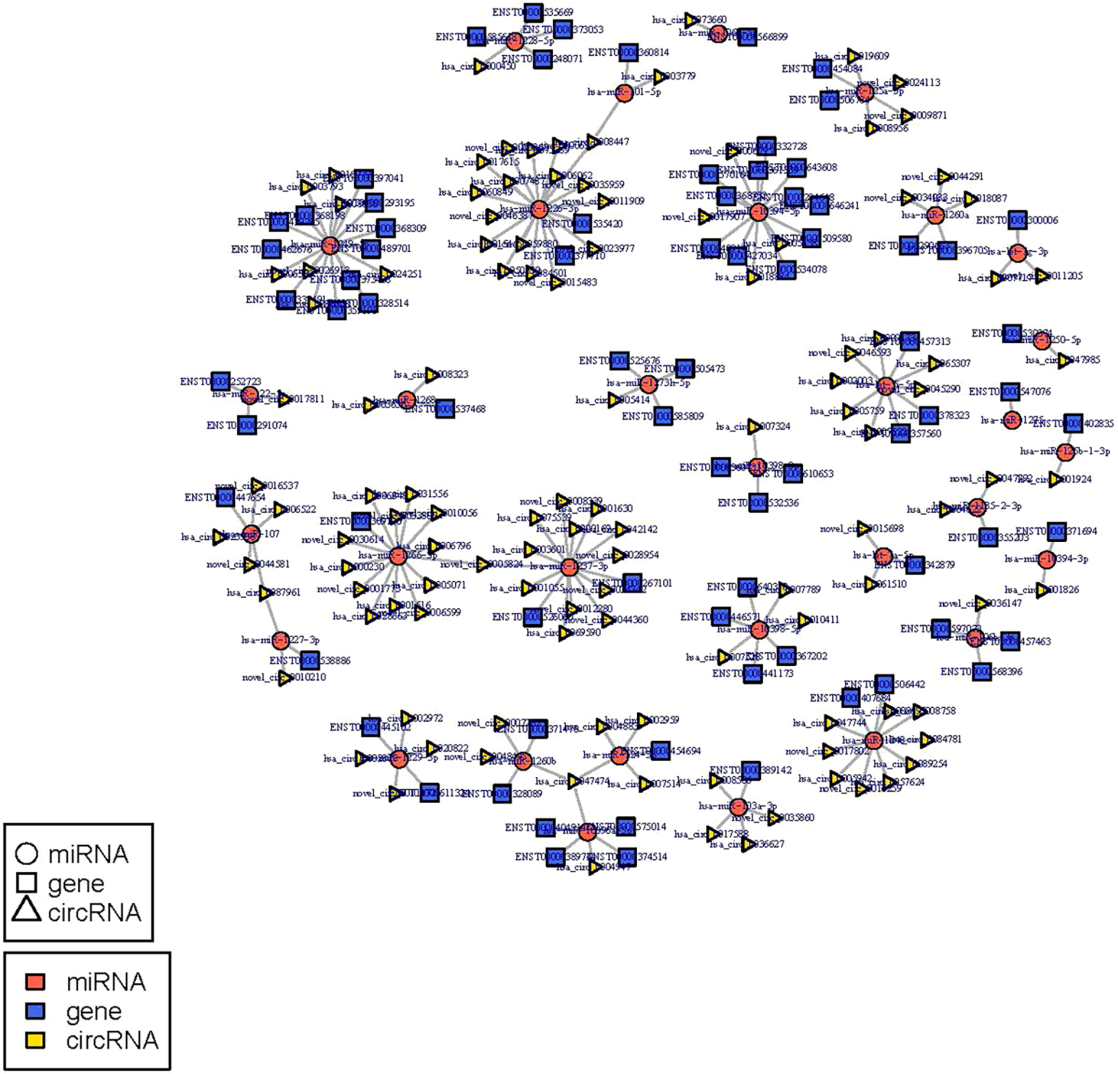
miRNA-circRNA-mRNA regulatory network: Red circle node represents miRNA, blue rectangle represents mRNA, and yellow triangle represents circRNA.

**Figure 6:**
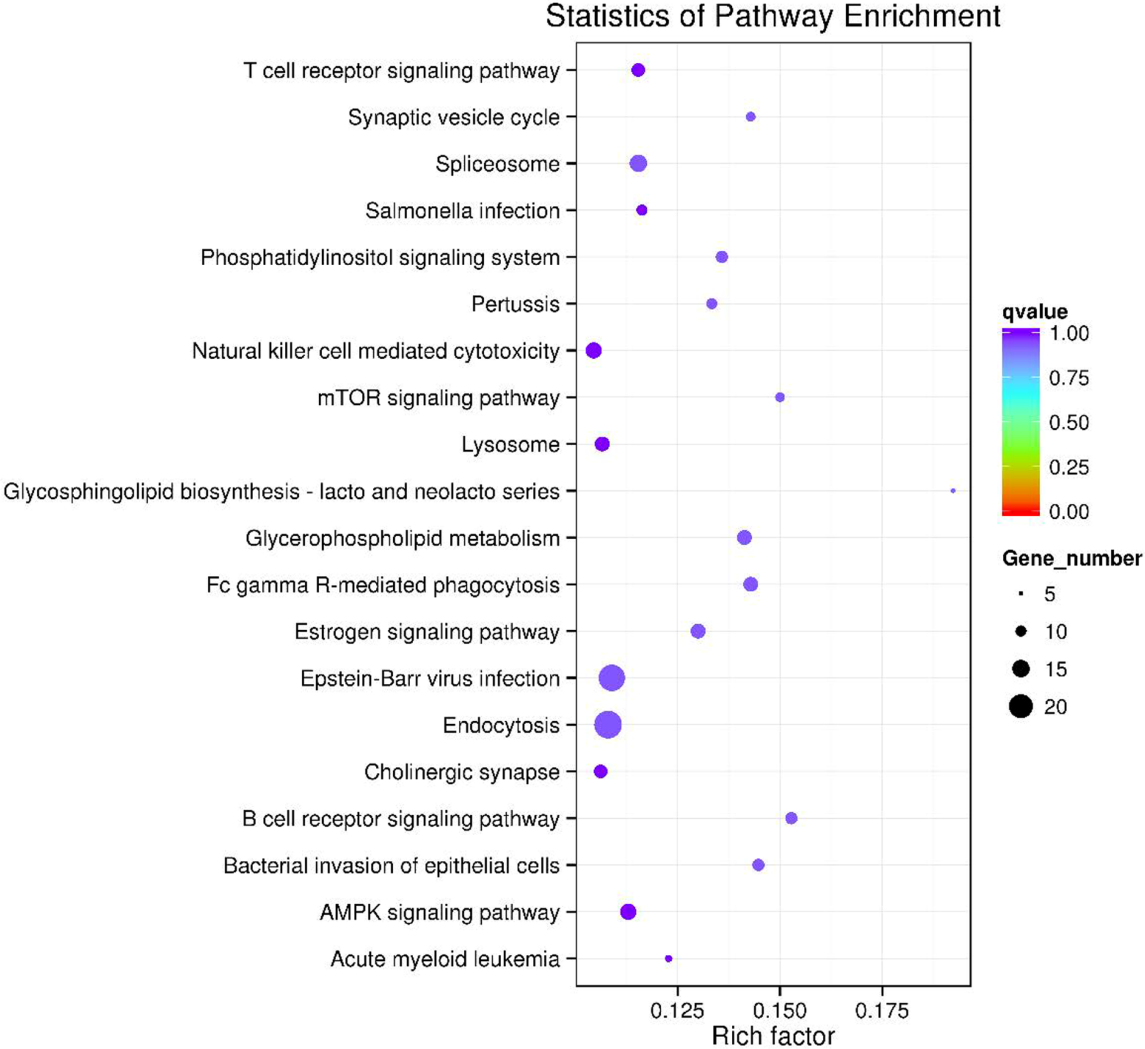
The top 20 enriched KEGG pathways of key circRNA in ceRNAs.

The circRNA-miRNA-mRNA regulatory network was constructed with 232 nodes, including 32 miRNAs, 123 circRNAs, and 77 mRNAs (Fig. 5). GO and pathway enrichment analyses implied that the circRNAs in this network mainly participated in catabolic processes and critical signaling pathways (Fig. 6). Next, we found that hsa_circ_0042409, one of the top key circRNAs, regulated the expression of KLC1 by inhibiting miRNA hsa-miR-195-5p. Also, other ceRNA networks, such as circRNA hsa_circ_0003990, hsa-miR-6506-5p, and P2RX5 mRNA, were identified.

### qPCR experiment validation

**Figure 7:**
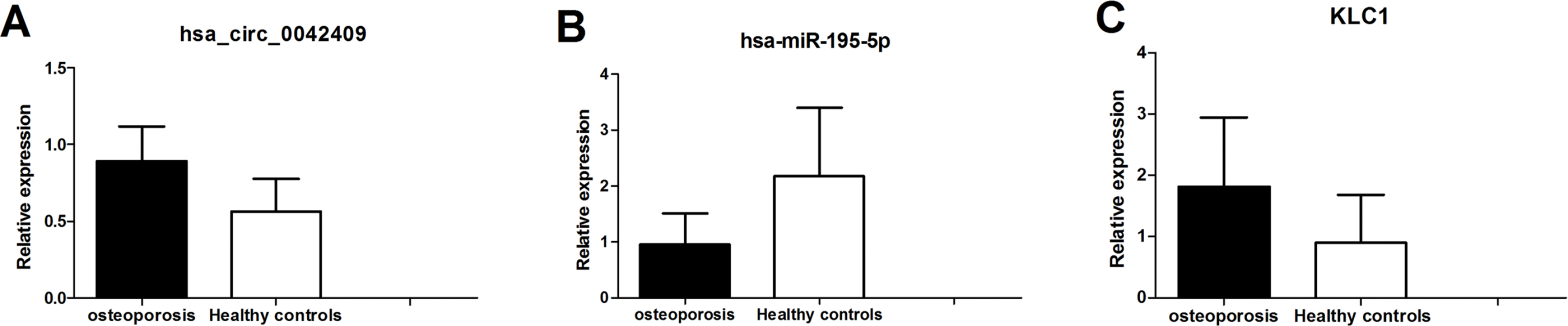
qPCR experiment validation of hsa_circ_0042409 (A), *P* < 0.05, hsa-miR-195-5b (B), *P* < 0.05 and KLC1 (C), *P* < 0.05.

The circrNA-mirNA-mrna regulatory network was constructed, and several key circRNAs related to miRNA and mRNA. Among these, hsa_circ_0042409 was linked to 7 miRNAs and 26 mRNAs, and KLC1 expression was regulated by inhibiting miRNA hsa-mir-195-5p. qPCR showed that the expression level of circRNA hsa_circ_0042409 and *KLC1* mRNA was significantly increased in male osteoporosis patients, while that of hsa-miR-195-5p was significantly decreased with the cutoff of *P*-value < 0.05 (Fig. 7) compared to healthy controls.

## Discussion

Osteoporosis (OP) is a systemic disease with reduction in bone mass and deterioration of microstructure augmenting the risk of fragility and susceptibility to fracture, especially in the spine, hip, and wrists. Increasing evidence has revealed that ncRNAs participated in various diseases by directly or indirectly regulating the corresponding gene expression. Furthermore, ncRNAs are associated with various diseases through indirect or direct regulation of the corresponding gene expression (Yang et al., 2018). ncRNAs, including miRNA, long lncRNA, and circRNA, play a crucial role in the occurrence, development, and progression of cancer (Dou et al., 2016). Importantly, previous studies have suggested that circmiRNAs may be used as vital biomarkers for osteoporosis (Fu et al., 2018; Materozzi et al., 2018). Wei et al. (2012) found that miR-34s inhibit osteoblast proliferation and differentiation in mice by targeting SATB2. Xia et al. (2016) also discovered that miR-31-5p and miR-424-5p were downregulated in cartilage-derived mesenchymal stem cells (CMSCs) from the degraded cartilage. Moreover, Yin et al. (2018) reported previously that circRUNX2 regulated RUNX2 to prevent osteoporosis via hsa-miR-203.

**Table 3:** GO enrichment of differentially expressed circRNAs.

GO_accession	Description	Term_type
GO:0043229	intracellular organelle	cellular_component
GO:0032991	macromolecular complex	cellular_component
GO:0043226	organelle	cellular_component
GO:0043227	membrane-bounded organelle	cellular_component
GO:0008152	metabolic process	biological_process
GO:0044237	cellular metabolic process	biological_process
GO:0065007	biological regulation	biological_process
GO:0050794	regulation of cellular process	biological_process
GO:0005488	binding	molecular_function
GO:0005515	protein binding	molecular_function

Over_represented_pValue	Corrected_pValue	CAD_item	CAD_list	Bg_item	Bg_list
1.02E−09	6.29E−06	266	317	12702	19499
1.30E−09	6.29E−06	143	317	5,321	19,499
2.55E−09	9.82E−06	273	317	13,290	19,499
1.85E−07	0.000397	253	317	12,437	19,499
8.42E−05	0.036064	231	317	11,961	19,499
0.000108	0.041014	215	317	10,723	19,499
0.00035	0.095557	232	317	11,972	19,499
0.000454	0.11517	214	317	10,819	19,499
0.000515	0.1256	276	317	14,763	19,499
8.12E−05	0.036064	232	317	11,455	19,499

**Table 4:** KEGG enrichment of differentially expressed circRNAs.

Term	ID	Input number	Background number	*P*-Value	Corrected P-Value
Thyroid hormone signaling pathway	hsa04919	9	119	0.001632	0.281902
Endocytosis	hsa04144	12	213	0.003185	0.281902
Cell cycle	hsa04110	8	124	0.007126	0.420405
HIF-1 signaling pathway	hsa04066	7	106	0.010274	0.43612
Epstein-Barr virus infection	hsa05169	10	202	0.01522	0.43612
Fc gamma R-mediated	hsa04666	6	91	0.017073	0.43612
phagocytosis	hsa05211	5	66	0.017248	0.43612
Renal cell carcinoma	hsa04068	7	134	0.030622	0.677503
FoxO signaling pathway	hsa05221	4	57	0.04012	0.778022
Acute myeloid leukemia	hsa05132	5	86	0.043956	0.778022

Herein, we performed whole transcriptome sequencing and investigated the regulatory mechanisms and functions of non-coding RNAs, especially circRNAs in the pathogenesis of osteoporosis. A total of 398 circRNAs, 51 miRNAs, and 642 mRNAs were identified to be significantly differentially expressed in osteoporosis compared to healthy controls. Moreover, GO and KEGG enrichment analysis illustrated that the host genes of significantly differentially expressed circRNAs were mainly enriched in the regulation of cell cycle processes, such as BP, organelle part CC, protein binding MF, Toll-like receptor signaling pathway, TNF signaling pathway, and thyroid hormone signaling pathway. Based on the circRNA-miRNA-mRNA regulatory network, some key circRNAs in osteoporosis were discovered further, such as hsa_circ_0042409, hsa_circ_0001924, hsa_circ_0003990, and hsa_circ_0000983.

hsa_circ_0042409 was linked to 8 miRNAs and 26 mRNAs in the ceRNA regulatory network, and was upregulated in osteoporosis. In addition, it regulated the expression of KLC1, RNH1, CPEN1, and STXBP2 by inhibiting hsa-miR-195-5p, hsa-miR-30b-5p, hsa-miR-32b-5p, hsa-miR-378d, hsa-miR-424b-5p, and hsa-miR-6763-5p. Qu, Chu & Wang (2017) demonstrated that miR-195-5p suppresses osteosarcoma cell proliferation and invasion by suppressing naked cuticle homolog 1. Zhang et al. (2019) discovered that DOC2B promoted insulin sensitivity in mice via a novel KLC1-dependent mechanism in skeletal muscle. Importantly, we found that circRNA hsa_circ_0042409 and *KLC1* mRNA were significantly increased while hsa-miR-195-5p was significantly decreased in male osteoporosis patients by whole transcriptome sequencing and further validated by qPCR. These findings coincidentally suggested that key circRNA, hsa_circ_0042409, is associated with the development of osteoporosis. The increased expression of hsa_circ_0042409 regulated the expression level of KLC1 by spongy hsa-mir-195-5P, thus promoting the pathogenesis of osteoporosis.

## Conclusions

Although further studies might be needed to support these findings, key cicrRNAs (hsa_circ_0042409 et al.) play a major role in the pathogenesis of osteoporosis and could be used as potential biomarkers or targets in the diagnosis and treatment of osteoporosis.

##  Supplemental Information

10.7717/peerj.11420/supp-1 Supplemental Information 1 Sequencing data

## Additional Information and Declarations

### DNA Deposition

The following information was supplied regarding the deposition of DNA sequences: The RNA-seq data are available at NCBI GEO: PRJNA667727.

### Data Availability

The following information was supplied regarding data availability: The RNA-seq data are available at NCBI GEO: PRJNA667727 and the raw measurements are available in the Supplemental File.
